# Benzene Tetraamide: A Covalent Supramolecular Dual Motif in Dynamic Covalent Polymer Networks

**DOI:** 10.1021/acs.macromol.3c01083

**Published:** 2023-08-11

**Authors:** Huiyi Zhang, Annemiek van Hertrooij, Tobias Schnitzer, Yinjun Chen, Soumabrata Majumdar, Rolf A. T. M. van Benthem, Rint P. Sijbesma, Johan P. A. Heuts

**Affiliations:** † Institute for Complex Molecular Systems & Laboratory of Macromolecular and Organic Chemistry, Department of Chemical Engineering & Chemistry, Eindhoven University of Technology, PO Box 513, 5600 MB Eindhoven, The Netherlands; ‡ Laboratory of Physical Chemistry, Department of Chemical Engineering & Chemistry, Eindhoven University of Technology, PO Box 513, 5600 MB Eindhoven, The Netherlands; § PTX-C, Shell Technology Center Amsterdam, Grasweg 31, 1031 HW Amsterdam,The Netherlands

## Abstract

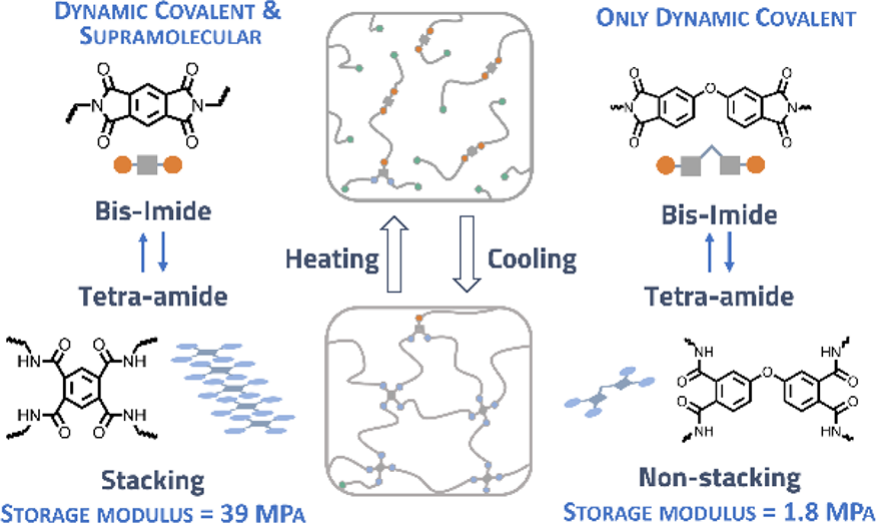

In dynamic polyamide networks, 1,2,4,5-benzene tetraamide (B4A) units act simultaneously as a dynamic covalent cross-linker and as supramolecular stacking motif. This results in materials with a rubbery plateau modulus that is about 20 times higher than that of a corresponding reference network in which the supramolecular interaction is suppressed. In branched polyamides with the same B4A dynamic motif, hydrogen bonding and stacking lead to strong and reversible supramolecular networks, whereas a branched polyamide with the nonstacking reference linker is a viscous liquid under the same conditions. Wide-angle X-ray scattering and variable-temperature infrared experiments confirm that covalent cross-linking and stacking cooperatively contribute to the dynamics of the network. Stress relaxation in the reference network is dominated by a single mode related to the dynamic covalent chemistry, whereas relaxation in the B4A network has additional modes assigned to the stacking dynamics.

## Introduction

**Scheme 1:**
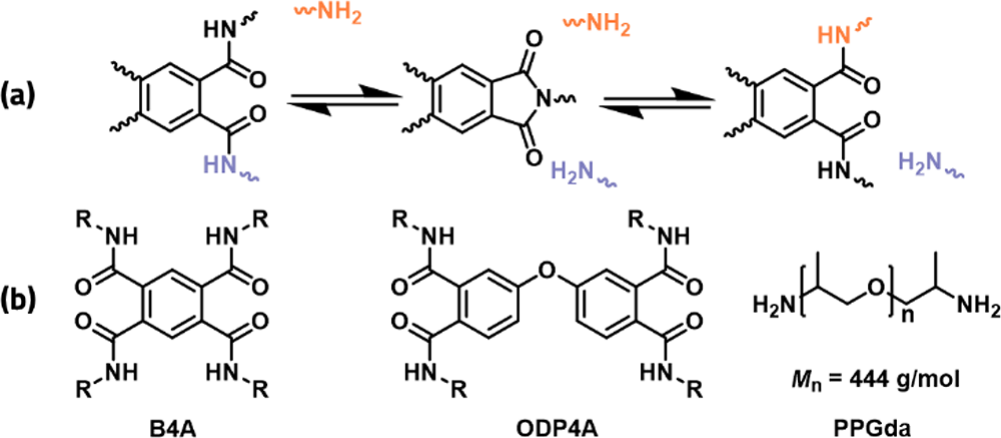
(a) Transamidation of Internally Catalyzed Polyamide Networks via Bisamide-Imide Equilibrium and (b) Structures of Cross-linking and Monomer Units

The incorporation of dynamic covalent and supramolecular motifs in polymers is influential in recent developments in polymer science. Both endow polymers with dynamic features that create responsiveness and improve processability and recyclability. Dual dynamic networks involving a combination of reversible covalent and noncovalent interactions have been demonstrated with various designs and show tunable responsiveness and self-healing properties, providing a new strategy to manufacture a wide range of dynamic synthetic polymers.^1−8^ However, the construction of such systems often requires multiple functional groups as dynamic motifs.^9^ In a recent paper, we presented a new covalent adaptable polymer network based on the bisamide-imide equilibrium in 1,2,4,5-benzene tetraamide (B4A) derivatives.^10^ At the same time, the group of Du Prez reported dynamic polyamide networks based on the bisamide-imide equilibrium in other diamides.^11^ The B4A-based dynamic polyamide network shows fast relaxation kinetics at relatively low temperatures (<140 °C) and easy (re)processability yet requires little synthetic effort using commercially available compounds. Transamidation in this system is dissociative in nature with a relatively stable imide as the intermediate (Scheme 1a), and this leads to a gel-to-sol transition at higher temperatures. In our previous study, the temperature dependence of the storage and loss moduli was consistent with an abrupt reduction in cross-link density at elevated temperatures, which we tentatively ascribed to the combined effect of the dissociation of chemical linkages and hydrogen-bonded stacks.^10^ In the current work, we establish the effect of supramolecular stacking on the mechanical properties and the dynamics of the polyamide network by comparing chemical networks in which B4A and oxidiphenyl tetraamide (ODP4A, a nonstacking reference linker) are used. Additionally, their branched polyamide derivatives are synthesized to study the supramolecular network in the absence of covalent cross-links. We use wide-angle X-ray scattering (WAXS) and variable-temperature infrared spectroscopy (VT-IR) to establish structural and chemical consequences of stacking in the network, and stress relaxation and dynamic mechanical thermal analysis (DMTA) are used to study the rheological profiles of the polyamide networks.

## Experimental and Computational Details

### Materials

Reagents and solvents were used as received without purification, unless otherwise stated. Poly(propylene glycol) bis(2-aminopropyl ether) (PPGda, *M*_n_ = 444 g/mol) and pyromellitic dianhydride (PMDA, 97%) were purchased from Sigma-Aldrich. 4,4′-Oxydiphthalic anhydride (ODPA, 99%) was purchased from Fluorochem. Tetrahydrofuran (THF, 99.8%) was purchased from Biosolve and kept dry over 4 Å molsieves.

### Synthesis of Polyimides

In a typical reaction, 10 g of dry PPGda (0.0225 mol) was added to a 100 mL round-bottom flask equipped with a stirring bar and kept under argon flow, followed by the addition of 4.910 g (0.0225 mol) of PMDA dissolved in 30 mL of anhydrous THF. The reaction mixture was left to reflux overnight, followed by the removal of solvent. The mixture was then transferred to a vacuum oven at 110 °C for further imidization overnight to yield a waxy oil as the product. Conversion of the amic acid groups into imides was confirmed by FT-IR. Similar conditions were used using ODPA.

### Synthesis of Covalent Adaptable Polyamide Networks

In a typical reaction for the synthesis of the covalent adaptable polyamide networks, 3 g of polyimide was introduced to a 50 mL round-bottom flask equipped with a stirring bar and kept under argon flow, followed by the addition of another equivalent of PPG-diamine (∼2.128 g, 1 equiv with respect to the PPGda in the polyimide). To the flask, 10 mL of anhydrous THF was added, and the mixture was left to reflux to obtain a homogeneous mixture. After 4 h, solvent was removed under reduced pressure, and the resulting viscous mixture was then transferred to a vacuum oven for further curing at 80–100 °C for 24–48 h. FT-IR spectra were obtained to monitor the conversion of imide to amide groups.

### Synthesis of Branched Polyamides

In a typical reaction to synthesize branched polyamides, 3 g of polyimide was introduced to a 50 mL round-bottom flask equipped with a stirring bar under argon flow, followed by the addition monofunctional Jeffamine M-600 (∼7.75 g, 1 equiv with respect to the imide group in polyimide). To the flask, 10 mL of anhydrous THF was added, and the mixture was left to reflux to obtain homogeneous mixture. After 4 h, solvent was removed under reduced pressure, and the resulting viscous mixture was then transferred to a vacuum oven for further curing at 80 °C for 24–48 h. FT-IR spectra were taken to monitor the conversion of imide to amide groups.

### Gel Contents and Swelling Ratios

For gel content determinations, approximately 40 mg of the dry network was weighed and swollen in 15 mL of a THF or methanol/THF (50/50 v/v) mixture and kept for 3 days at room temperature. Subsequently, the sample was filtered and dried in a vacuum oven at 80 °C for 24 h. The gel content was determined using eq 1:
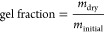
1where *m*_initial_ is the mass of the sample before extraction and *m*_dry_ is the mass after extraction and drying.

For the swelling ratio tests, approximately 40 mg of the dry network was weighed and swollen in 20 mL of THF and kept at room temperature for 3 days. The sample was then removed from the solvent, gently tapped with dry filter paper, and weighed again. The swelling ratio was determined using eq 2:
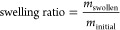
2where *m*_initial_ is the mass of the dry sample before the swelling test and *m*_swollen_ is the mass of the swollen sample.

### Compression Molding

The materials were compression molded at 120–130 °C under a pressure of 100 bar for 30 min in a Collin Press 300G and subsequently cooled with water.

### Dynamic Mechanical Thermal Analysis

Compression molded samples (ca. 12.0 × 10.0 × 0.8 mm) were measured on a DMA Q850 (TA Instruments) with a film tension setup. A temperature ramp, typically from −60 to 200 °C, was programmed with a heating rate of 3 °C·min^–1^ at a frequency of 1 Hz. However, due to the softening and elongation of the samples at higher temperature, the data acquisition stopped when the clamp reached the motion limit. Preconditioning for 30 min at the starting temperature, a preload force of 0.01 N, an amplitude of 10 μm, and a force track of 110% were used. The storage and loss moduli were recorded as functions of temperature. The glass-transition temperature was determined from the peak maximum of tan(δ).

### Shear Rheology

All rheological measurements on the networks were carried out on a DHR 20 rheometer equipped with an ETC oven setup (TA Instruments) with parallel plate geometry. Typical samples for rheology measurements were approximately 8 mm in diameter and 1 mm in thickness, prepared by compression molding of the dry product at 120–130 °C and a pressure of 100 bar for 30 min. For all measurements on the covalent adaptable polyamide networks, a constant normal force of 1 ± 0.5 N was applied by automatically adjusting the gap to ensure proper contact between the sample and plates. Prior to each measurement, samples were conditioned for 2 h at the highest measurement temperature (i.e., 170 °C for the B4A-based network and 120 °C for the OPD4A-based network) to ensure the full establishment of the equilibrium state at each temperature. Stress relaxation measurements for all samples were conducted at a step strain of 1%. The relaxation modulus as a function of time *G(t)* was monitored over time at a range of temperatures. For the B4A-based and ODP4A-based polyimides, parallel plate geometries of 8 mm in diameter were used, the samples were loaded as viscous liquids using a spatula, and the gap was set to 0.8 mm to ensure proper contact between the sample and plates. A temperature ramp was carried out from 25 to 150 °C at a heating rate of 3 °C·min^–1^ with a frequency of 1 Hz and strain of 1%.

### Thermogravimetric Analysis

Thermogravimetric analyses were performed on a TGA Q500 instrument from TA Instruments under a N_2_ rich atmosphere. Samples were heated from 30 to 600*°*C at a rate of 10 °C·min^–1^. Temperature calibration was performed using the Curie points of high purity aluminum, nickel, and perkalloy standards.

### Differential Scanning Calorimetry

Differential scanning calorimetry (DSC) measurements were performed on a TA Instruments Q2000 differential scanning calorimeter equipped with an RCS90 cooling accessory using aluminum hermetic pans. For each measurement, 5–10 mg of sample was used. The sample was scanned twice from −80 to 200 °C at a heating rate of 20 °C·min^–1^, followed by a cooling cycle in the same temperature range at a rate of 20*°*C·min^–1^. *T*_g_ was determined as the midpoint of the step in the heat flow curve and analyzed using TA Universal Analysis software.

### Variable-Temperature Infrared Spectroscopy

Variable-temperature infrared (VT-IR) spectra were recorded on a JASCO Tensor 27 with a Pike ATR temperature unit. For each measurement, the sample was scanned from 30 to 180 °C at a heating rate of 3 °C·min^–1^ under N_2_ flow. Background spectra at varying temperatures were recorded under the same conditions and subtracted from the recorded raw spectra using OriginLab to obtain the final spectra.

### Electronic Structure Calculations

Computational analyses of the monomers and dimers were performed using Schrödinger Maestro Suite 2021–2. The input structures were generated using the built-in 2D structure generator followed by geometry minimization with Macromodel (OPLS4 force field, vacuum). The dimer structures of B4A were oriented such that linear hydrogen bonds along the helical screw axes are formed. For the OPD4A-based tetraamide, no such geometry could be obtained due to steric restrictions; thus, a conformational search (OPLS4 force field, vacuum, 5000 steps) was performed and the lowest energy structure was used as starting structure. Geometry optimization of the structures was performed using Jaguar at the B3-LYP-D3/6-311G+** level of theory (vacuum). Frequency calculations on the optimized structures yielded no imaginary frequencies, indicating stationary points on the potential energy surface.

## Results and Discussion

**Figure 1:**
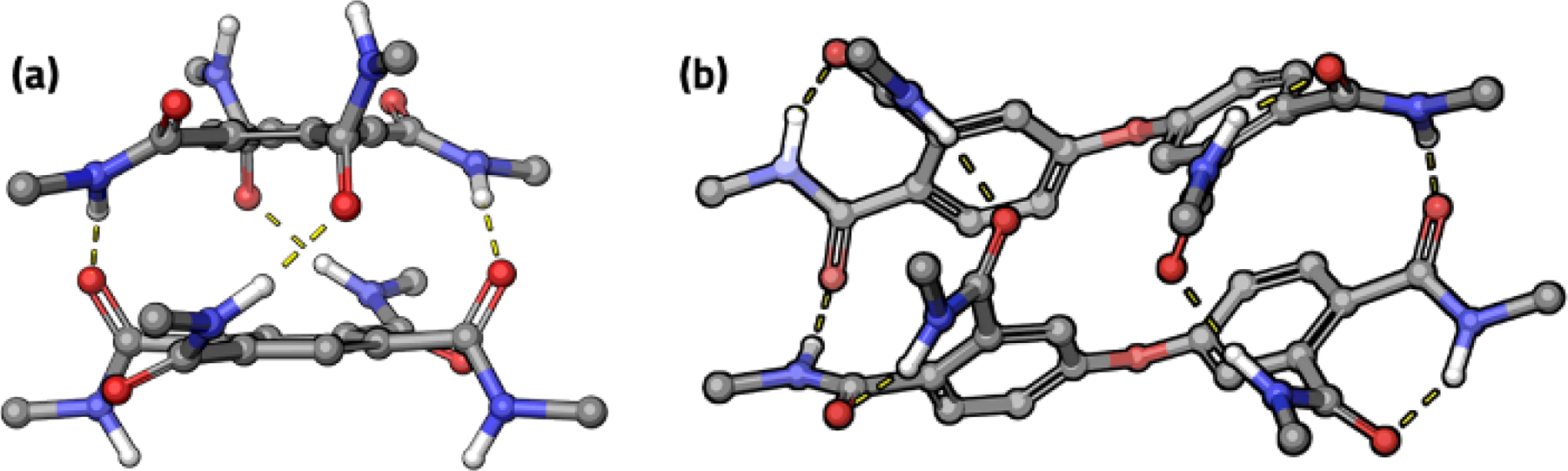
DFT optimized geometries (side view) of dimers of (a) B4A and (b) ODP4A.

First, stacking properties of B4A and reference cross-linker ODP4A were investigated with density functional theory (DFT) calculations on monomers and dimers (see the Supporting Information for more details). In the B4A-dimer, the molecules are connected via four hydrogen bonds (Figure 1a). Four free N–H donor and four free carbonyl acceptor sites in the dimer are oriented such that hydrogen bonding along helical screw axes promotes further stacking in 1D columns. This geometry is in line with the reported formation of columnar stacks in the mesophase of benzene 1,2,4,5- tetradecanamide.^12^ The helical hydrogen bonding motif is similar to that in well-studied benzene triamide stacks^13^ where aggregation is strongly cooperative.^14^ In the optimized geometry of the ODP4A dimer (Figure 1b), eight hydrogen bonds are formed within the dimer that satisfy all donor and acceptor sites, disfavoring further aggregation.

**Scheme 2:**
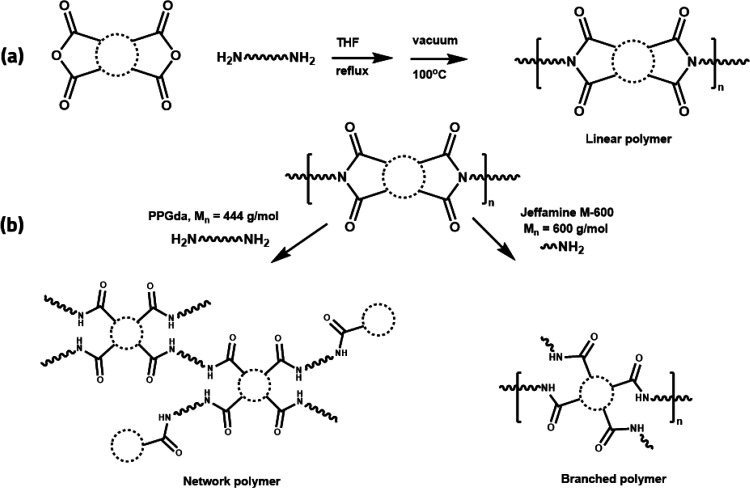
Synthesis of (a) Polyimides and (b) Covalent Adaptable Polyamide Networks and Branched Polyamides using PPGda or Monofunctional Jeffamine M-600

For a comparative study of the interplay between supramolecular stacking and dynamic covalent chemistry, two covalent adaptable polyamide networks and two branched polyamides based on B4A and ODP4A were prepared. In a two-step synthetic strategy,^10^ first, a linear polyimide was synthesized via the polycondensation reaction of poly(propylene glycol) bis(2-aminopropyl ether) (PPGda, *M*_n_ = 444 g/mol) with a dianhydride (see Figure S1 for DMTA results), followed by the ring opening of imide groups either via further addition of PPGda to obtain the covalent networks or addition of monofunctional Jeffamine M-600 (*M*_n_ = 600 g/mol) to obtain the branched polyamides (Scheme 2).

The B4A- and ODP4A-based networks were compression molded for further characterization. The gel content and swelling ratios of the covalent adaptable networks were measured by extraction of the sol phase in two different solvent systems, namely, tetrahydrofuran (THF) and a hydrogen-bond breaking solvent mixture of THF and methanol. The measured gel content of the B4A-based network dropped from 0.97 to 0.92 when the solvent was changed from THF to THF/methanol, while for the ODP4A-based networks, the gel contents in these solvent systems were equal within experimental errors (0.86 and 0.85, respectively, see Table S1 for more details). These results are consistent with the existence of additional physical cross-links in the B4A-based network and their absence in the ODP4A-based network.

**Figure 2:**
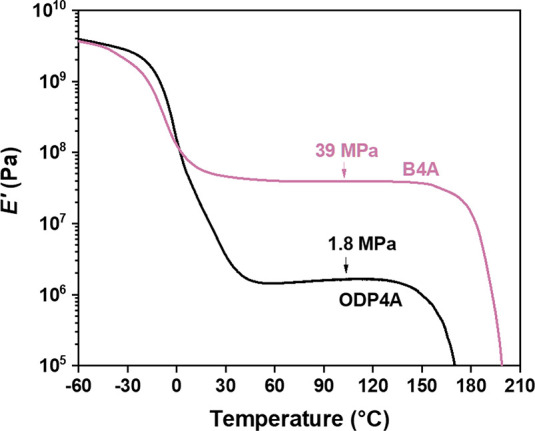
Dynamic mechanical thermal analysis of dynamic covalent B4A-based and ODP4A-based polyamide networks at a heating rate of 3 °C·min^–1^ from −60 to 200 °C and a frequency of 1 Hz.

The effect of supramolecular interactions on the modulus in these networks was investigated with dynamic mechanical thermal analysis (DMTA), and the results are shown in Figure 2. Both B4A-based and ODP4A-based networks show a glass transition around 0 °C (see Figure S2 for more details), followed by a prolonged rubbery plateau up to 150 °C. Both networks show a drastic drop in *E′* upon heating above 150 °C, ascribed to a reduction in cross-link density due to a shift of the bisamide-imide equilibrium, similar to what was found in our previous paper on B4A dynamic covalent networks (see Scheme 1 and Figure S2 for more details).^10^ However, there is a striking difference between the plateau rubbery moduli *E′* in the B4A and the ODP4A systems. The B4A-based network has a modulus that is more than 20 times higher than that of the ODP4A-based network (39 and 1.8 MPa, respectively). The higher plateau value in the B4A network can only be partially explained by its higher covalent cross-link density implied by the higher gel content (see above). We attribute most of the difference in *E*′** to the presence of hydrogen-bonded stacks in the B4A-based network and their absence in the ODP4A-based network. A high aspect ratio of these stacks, stiffness, and a high volume fraction of up to 25% (based on the fraction of PMDA in the network) results in efficient stiffening. Between the *T*_g_ and approximately 150 °C, a part of the difference in *E*′** may be ascribed to differences in the amide/imide equilibrium between the networks (see VT-IR results discussed below).

To further examine the effect of supramolecular interaction on the mechanical properties of the polyamides, branched polyamides were prepared by reacting the PMDA and ODPA-based precursor linear polyimides with a monoamine rather than a diamine (see Scheme 2). Both products were completely soluble in THF. However, whereas the branched ODP4A-based polyamide is a viscous liquid at room temperature, the branched B4A-based polyamide is a solid that can be compression molded at 125 °C (for details, see the Supporting Information, Figures S5 and S6).

**Figure 3:**
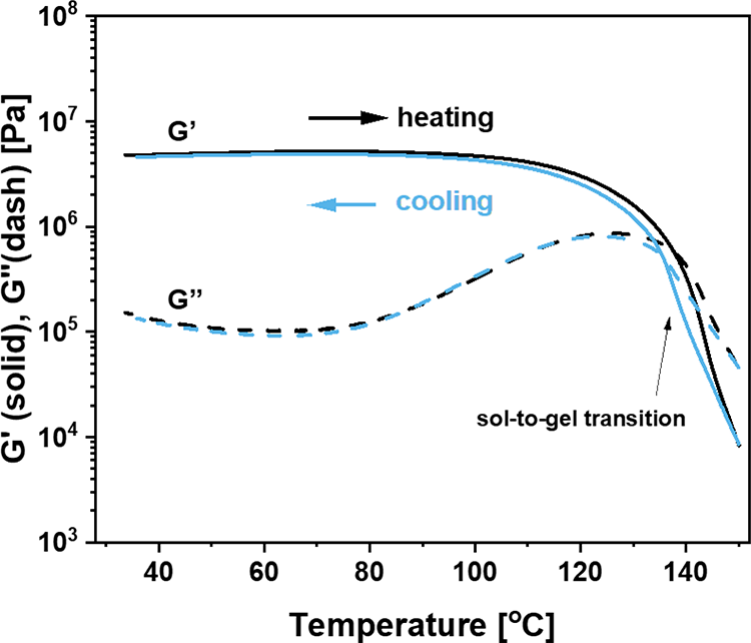
Temperature sweep results of branched B4A-based polyamide using oscillatory shear rheology with a heating and cooling rate of 3 °C·min^–1^ from 30 to 150 °C and a frequency of 1 Hz.

In Figure 3, the results of a temperature sweep in oscillatory shear rheology are shown for the branched B4A-based polyamide. This solid material is predominantly elastic (*G′* > *G″*) from room temperature up to about 130 °C. As no covalent cross-links are present in this soluble polymer, the rubbery behavior derives from physical cross-links.

Notably, with a plateau modulus *G′* of about 5 MPa, the supramolecular cross-links in the branched B4A polymer bring about a higher plateau modulus than the covalent cross-links in the ODP4A-based covalent network (for which a shear modulus *G′* ∼ 0.6 MPa can be estimated from the value of *E′* in DMTA). Around 130 °C, a gel-to-sol transition is observed, probably as the combined result of the dissociation of the physical cross-links and a shifting bisamide-imide equilibrium. Disintegration of the supramolecular network was fully reversible as is evident from the full restoration of *G′* upon cooling with little hysteresis.

**Figure 4:**
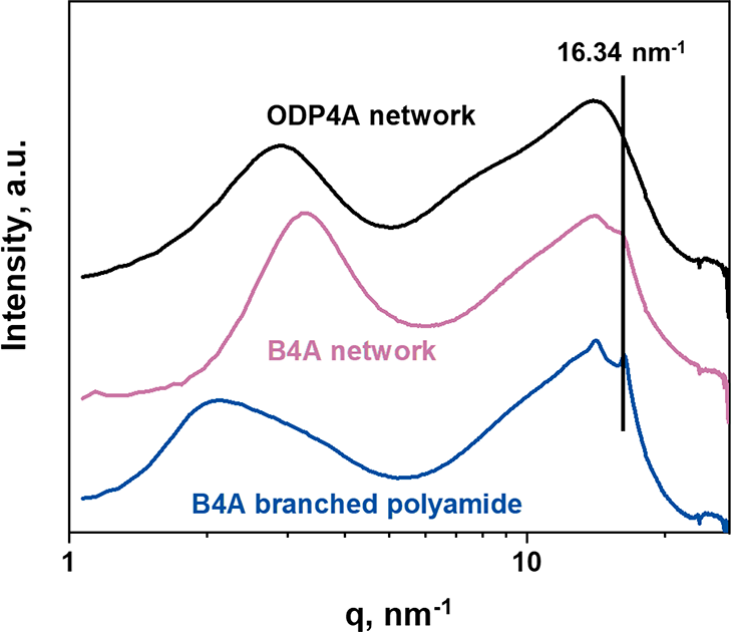
1D wide-angle X-ray diffractograms of the ODP4A (black) and B4A (purple) networks and the B4A branched polyamide (blue).

Stacking of the B4A motif in the covalent networks and the branched polymer was further investigated with wide-angle X-ray diffraction (WAXS). The diffractograms are shown in Figure 4. The dynamic covalent B4A-based network (pink line) and the branched polyamide (a supramolecular network, blue line) both have a diffraction peak corresponding to stacking of benzene rings with a distance of 0.38 nm, close to the distance of 0.40 nm found in the ordered columnar mesophase of benzene 1,2,4,5-tetradecanamide,^12^ but this peak is absent in the diffractogram of the ODP4A network (black line).

**Figure 5:**
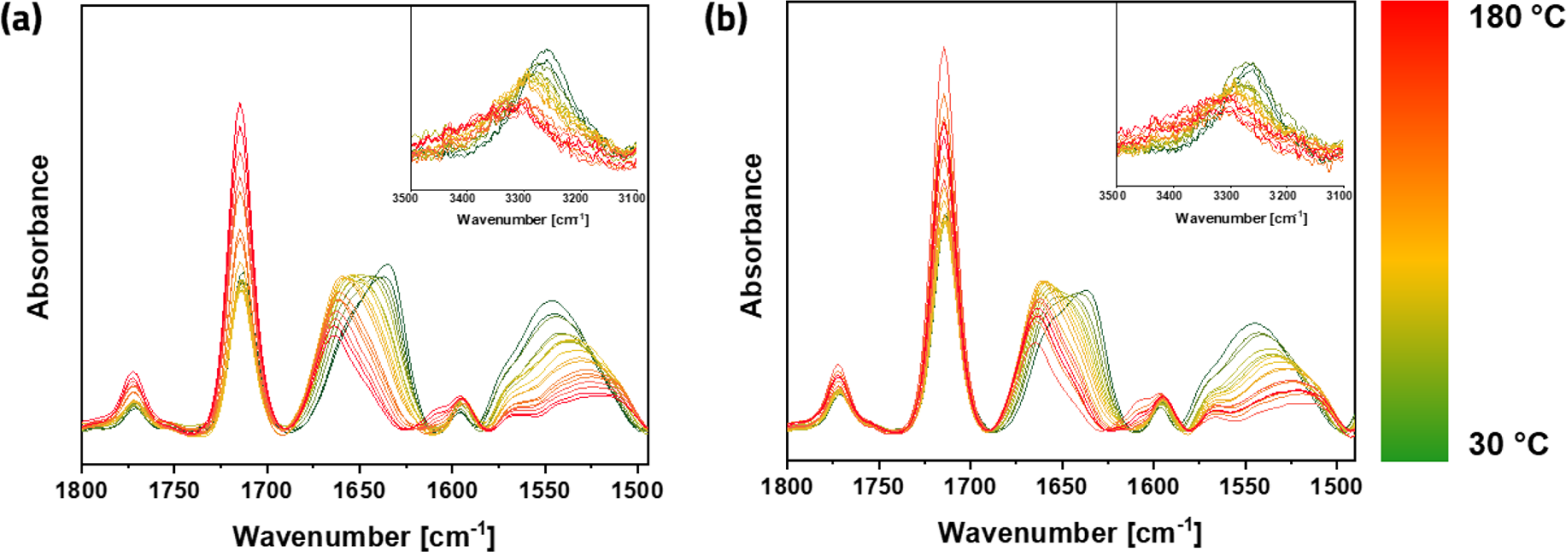
Carbonyl region in variable-temperature infrared (VT-IR) spectra of (a) B4A and (b) the ODP4A networks, with the −N–H region in inserts. Heating rate: 3 °C·min^–1^, from 30 °C (green) to 180 °C (red) with intervals of 10 °C.

Having clearly established the existence of stacking via intermolecular hydrogen-bonding of B4A tetraamide dynamic cross-links, we investigated the temperature dependence of the bisamide-imide equilibrium and hydrogen bonding in both the ODP4A and B4A networks with VT-IR spectroscopy in the temperature range from 30 to 180 °C. The results of these experiments are shown in Figure 5.

**Figure 6:**
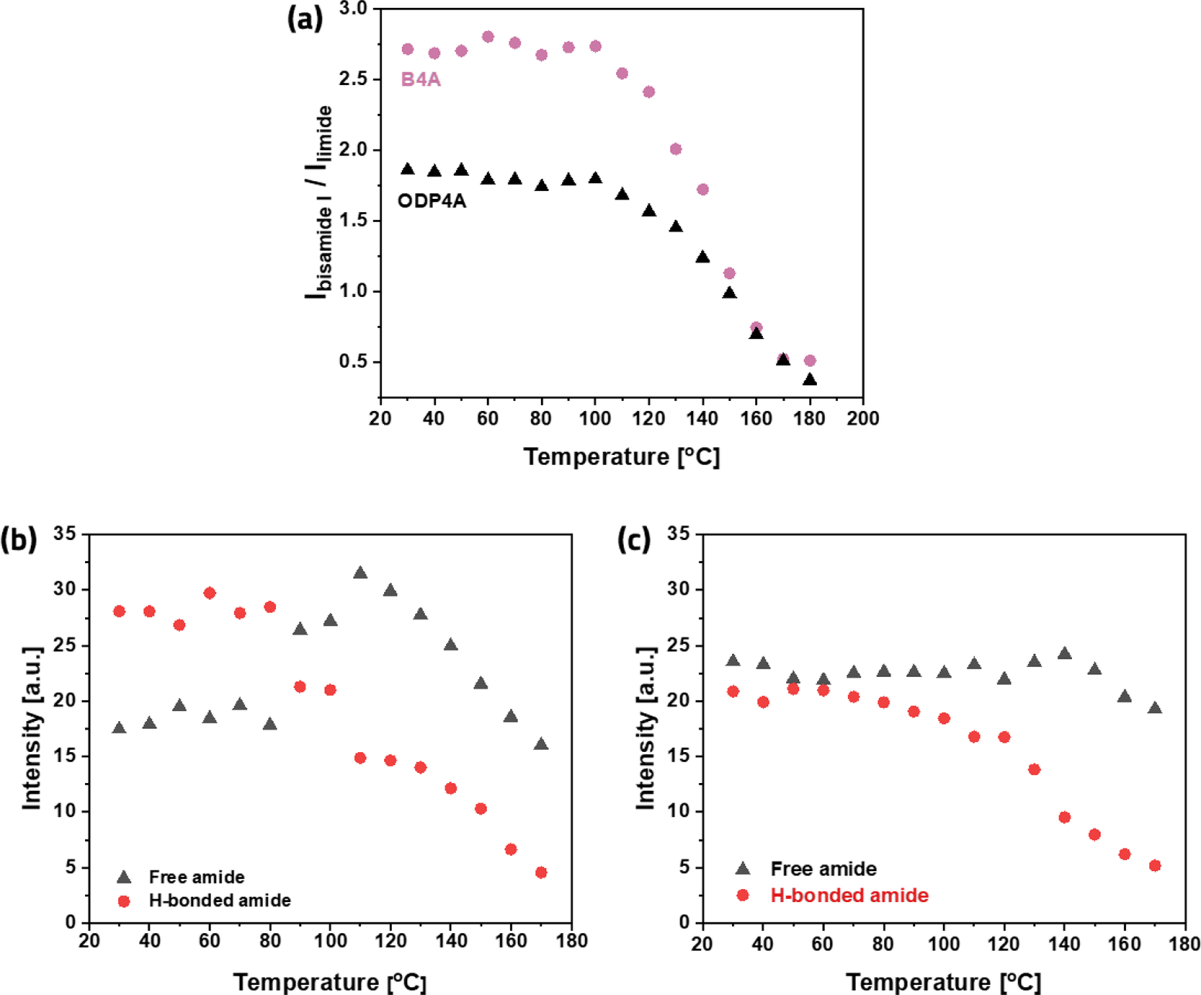
(a) Ratio of the intensities of amide I (∼1630 cm^–1^) and imide (∼1725 cm^–1^) peaks of B4A- and ODP4A-based polyamide networks. (b,c) Intensities of “free” amide I (1660 cm^–1^) and hydrogen-bonded amide I (1638 cm^–1^) obtained from peak deconvolution of amide I peak (1620–1680 cm^–1^) for (b) the B4A network and (c) the ODP4A network.

The temperature dependence of the amide-imide equilibrium was followed by comparing the intensities of the carbonyl bands of imides at 1725 cm^–1^ and those of amides at 1630 cm^–1^. In Figure 6a, the intensity ratio of these bands is plotted; up to a temperature of about 100 °C, this ratio is fairly constant, but it is significantly higher for the B4A network ((*I*_bisamide_/*I*_imide_ = 2.75) than for the ODP4A network (*I*_bisamide_/*I*_imide_ = 1.75). This implies that the equilibrium in the B4A-based material is shifted to the amide. Above 100 °C, the ratio drops steeply for both networks.

The onset of the shift in the equilibrium toward the imide precedes the drop in *E′* in DMTA by about 50 °C. From 150 to 180 °C, the bisamide/imide ratios are similar for B4A and ODP4A networks, indicating a similar covalent network density, while the elastic modulus remains much higher for the B4A network in this temperature range. This indicates that stacking via hydrogen bonds continues to contribute to the modulus of the B4A material up to 180 °C.

The extent of hydrogen bonding of the amides can be inferred from the positions of the amide I band (∼1630 cm^–1^), amide II band (∼1550 cm^–1^), and the N–H stretch band at 3310 cm^–1^. At 30 °C, the maxima of these bands are close to the values reported for strongly hydrogen bonded tetracarboxamide liquid crystals^12^ and benzene triamide thermoplastic elastomers.^15^ Shifts of amide bands and of the N–H stretch band at 3310 cm^–1^ (see insets of Figure 5) upon heating indicate that the hydrogen bonds between bisamide moieties in both B4A- and ODP4A-based networks begin to dissociate at higher temperatures. The amide I region was analyzed in more detail using peak deconvolution to quantify the shift from hydrogen-bonded to non hydrogen-bonded “free” amide with increasing temperature. The results of the analysis are shown in Figure 6b,c. In the B4A-based network, the intensities of both peaks remain constant up to a temperature of 90 °C at which there is a sudden decrease in hydrogen-bonded amides (and a corresponding rise in “free amides”); and both bands decrease steadily at temperatures above ∼110 °C, in line with the onset of the changing bisamide/imide ratio shown in Figure 6a. In the ODP4A-based network, where the DFT calculations suggest that much of the hydrogen bonds are intramolecular, the intensities do not show a sudden change: the band of the hydrogen-bonded amide steadily decreases from room temperature onward and the “free amide” band remains constant up to ∼140 °C.

**Figure 7:**
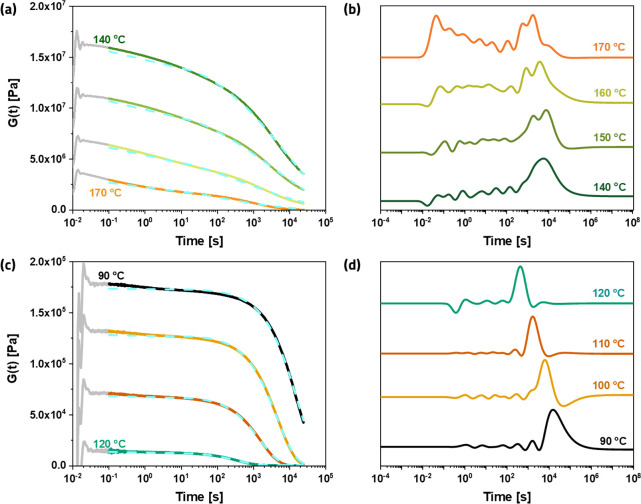
(a) Step-stress relaxation curves of a covalent adaptable B4A-based network at varying temperatures from 140 to 170 °C and (b) the corresponding relaxation spectra. (c) Step-stress relaxation curves of a covalent adaptable ODP4A-based network at varying temperatures from 90 to 120 °C and (d) the corresponding relaxation spectra. The relaxation curves in (a) and (c) were acquired using a step strain of 1%, and the dashed lines of both graphs represent the stretched exponential fitting using eq 3.

We investigated the dynamic nature of the B4A- and the ODP4A-based networks first with stress relaxation experiments. In Figures 7a,c, the relaxation moduli *G*(*t*) of both networks are shown for a range of different temperatures. Stresses in the networks are completely relaxed at the highest experimental temperatures, as expected for dynamic covalent networks.

Furthermore, the initial modulus of the B4A-based network at the highest temperature (170 °C) is more than 10 times higher than that of the ODP4A-based network at the lowest temperature (90 °C), and this is consistent with the DMTA results for both networks. Finally, a comparison of the stress relaxation profiles of the two systems shows that they are qualitatively different.

The relaxation behavior of neither system is adequately described by a single exponential (Maxwell model), and therefore, we used a stretched exponential function (eq 3) to fit the relaxation curves.^16−19^
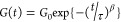
3

In this equation, *G*_0_ is the initial modulus, *t* is the time, τ is a characteristic relaxation time, and β is the stretch exponent, which is often considered as a parameter reflecting the dispersity of a particular relaxation mode. The results from fitting eq 3 to the data in Figure 7 are summarized in Table 1.

**Table 1:** Fit Parameters of Stress Relaxation Curves in Figure 7 using Eq 3

	B4A-based			ODP4A-based		
*T* (°C)	*G*_0_ (MPa)	β	τ (s)	*G*_0_ (MPa)	β	τ (s)
90				0.17	0.80	1.6 × 10^4^
100				0.13	0.84	4.5 × 10^3^
110				0.068	0.81	1.5 × 10^3^
120				0.013	0.74	3.4 × 10^2^
140	16	0.33	6.7 × 10^3^			
150	11	0.27	3.3 × 10^3^			
160	7.1	0.22	6.2 × 10^2^			
170	3.3	0.23	1.2 × 10^2^			

Relaxation of the ODP4A-based network is characterized by a relatively high and fairly constant value of ∼0.8 for β, close to that of the Maxwell model (β = 1). The very low values of fitted β of the B4A-based networks, varying from 0.33 to 0.23 with increasing temperature, indicate a very broad distribution of relaxation modes. This is more clearly seen from the relaxation spectra obtained using a generalized Maxwell model (see the Supporting Information for details),^20,21^ shown in Figures 7b,d for the B4A and ODP4A-based networks, respectively. The relaxation spectra of the ODP4A-based networks are relatively simple and clearly dominated by a single slow mode at high relaxation times (caused by the bond exchange reactions), which shifts to lower time scales (lower τ) with increasing temperature. The relaxation spectra of the B4A-based networks, however, are clearly more complex with faster modes in addition to the slow mode at relaxation times close to τ, and it is proposed that these faster modes are related to supramolecular exchange reactions. Finally, it is interesting to note that very similar values for *t* are obtained in the series of experiments on the B4A-based networks as in the ODP4A-based networks but at a temperature that is 50 °C higher than in the ODP4A-based network.

**Figure 8:**
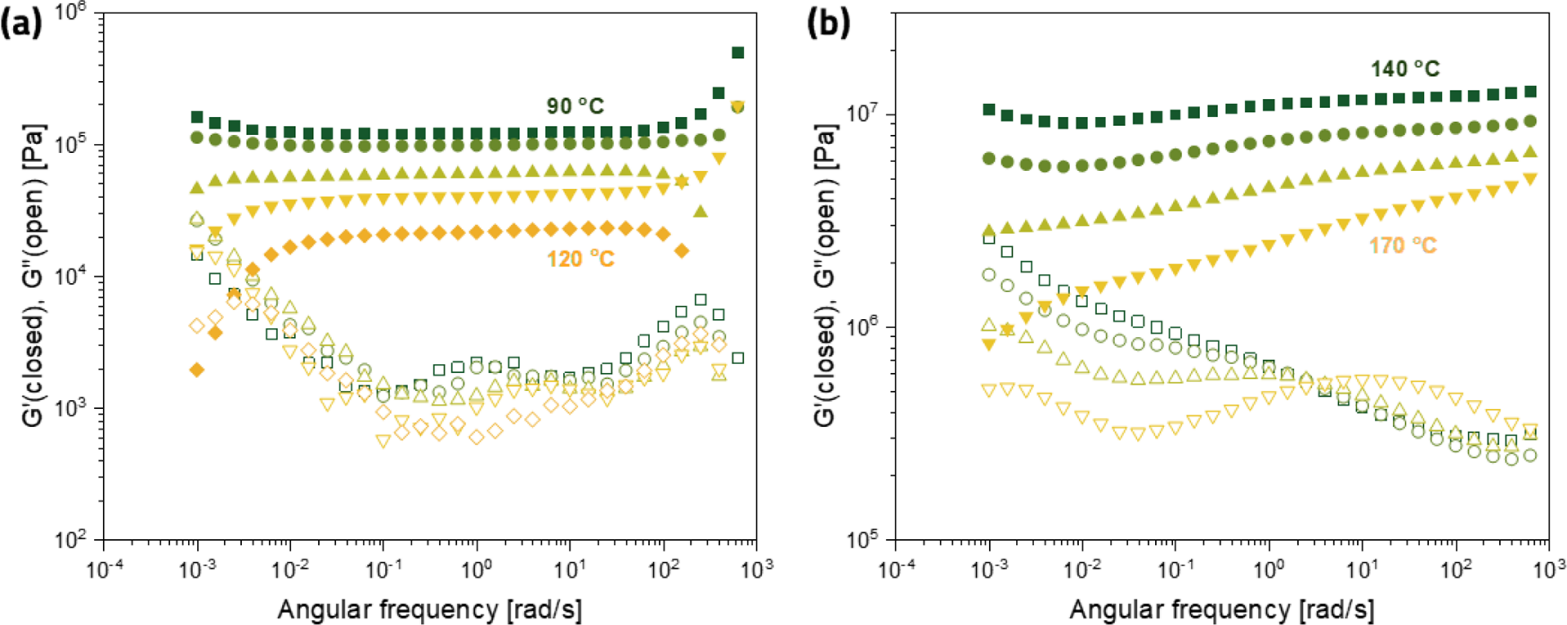
Frequency dependence of the storage (*G′*) and loss (*G*″) moduli for (a) the ODP4A-based network at a temperature range of *T* = 90–120 °C and (b) the B4A-based networks at a temperature range of *T* = 140–170 °C.

Finally, we studied the dynamic nature of the networks by oscillatory sweep experiments (ω = 10^–3^ – 628 rad·s^–1^) at a range of different temperatures. In Figure 8a, the storage and loss moduli of the ODP4A network are shown in the temperature range of 90 to 120 °C. From this figure, we can conclude that the behavior of this network is consistent with that of a stereotypical dissociative network:^22,23^ (i) we observe a fairly constant plateau modulus at temperatures at which the exchange reactions are not significantly operative and (ii) we observe a drop in modulus at very low frequencies implying a more liquid-like behavior caused by a significant occurrence of the exchange reaction above a certain temperature (the frequency at which this happens increases with increasing temperature). Here, this happens at *T* ≈ 110 °C and higher, and (iii) the plateau value of *G′* decreases with increasing temperature because of a shift in the equilibrium toward a more dissociated state. Overall, these results are consistent with the DMTA results in Figure 2 (measured at 1 Hz) and the infrared data in Figure 6a.

When we compare these results with those obtained for the B4A-based network in Figure 8b, we clearly see very different behavior. Even at temperatures of 140 and 150 °C, we do not observe the characteristic drop in *G′* at low frequencies. At 160 and 170 °C, we observe a decreasing *G′* with decreasing frequency and something that could be interpreted somewhat as a drop around 10^–2^ rad·s^–1^ at 170 °C. We do observe the overall decrease of *G′* at all frequencies with increasing frequency, which is consistent with a shifting equilibrium toward the more dissociated state. Overall, these results are conceivably explained by the fact that the exchange reactions become operative at lower temperatures, but that the supramolecular interactions hinder flow up to much higher temperatures, as is also shown in Figure 2.

## Conclusions

Extensive hydrogen bonding and stacking in the network with 1,2,4,5-benzentetracarboxamide as a dynamic motif result in a rubbery plateau modulus that is about 20 times higher than that of the corresponding ODP4A reference network. The results presented here clearly show that this large difference is caused by the fact that in the OPD4A network, and we only have the dynamic covalent cross-links, whereas in the B4A network polymer, the cross-linker additionally forms stacks. These stacks act just like uniform crystallizable hard segments in segmented copolymers and that stiffen the material more strongly than isolated cross-links.^24^ The picture that emerges from the combined experimental studies is consistent with the differences in the aggregation predicted by DFT modeling of the dimers. Variable-temperature infrared experiments confirm this interpretation. A remarkable feature in the VT-IR studies of both networks is that the onset of the shift in bisamide-imide equilibrium (at 100 °C) precedes the loss in modulus by 50 °C. An important factor that limits the loss in modulus when the equilibrium shifts is the fact that initially, conversion of amides to imides does not result in full loss of cross-links but to conversion of tetrafunctional to trifunctional diamide-imide cross-links. The VT-IR experiments also show that the amides in the B4A network are thermodynamically stabilized by stacking, as is evident from the higher bisamide/imide intensity ratios compared to the ODP4A network. However, in the B4A network, hydrogen bond dissociation already starts at 90 °C when B4A stacks begin to dissociate. We interpret the persistence of a high modulus above 90 °C as a result of the cooperativity of the stacking, a supramolecular polymerization process that is characterized by long stacks coexisting with significant amounts of monomeric units.^25^ Therefore, even after the onset of the dissociation of the long stacks at 90–100 °C, 1D aggregates remain present and keep the modulus higher than in the ODP4A network. From around 150 °C, due to dissociation of the stacks in the B4A network, the amide-imide equilibrium shifts quickly to reach the same value as in the ODP4A network. At this stage, the modulus of the B4A network is still higher than that of the ODP4A network because of the large effect of the remaining stacks of tetraamide.

Our comparative study demonstrates how a single molecular unit -B4A- leads to cooperative dynamics of reversible covalent bonds and supramolecular interactions in a covalent polymer network.

## Supporting Information Available

Reaction schemes relating to syntheses, procedure for determining relaxation spectra, DFT results, DMTA results, swelling ratios and gel contents, TGA and DSC results, images and rheological data of ODP4A- and B4A-based polyamides, and FT-IR spectra PDF
