# Supplementary material for: Benzene Tetraamide: A Covalent Supramolecular Dual Motif in Dynamic Covalent Polymer Networks
Source: Macromolecules. 2023 Aug 11;56(16):6452–60. doi: 10.1021/acs.macromol.3c01083 (PMC10448746; doi:10.1021/acs.macromol.3c01083)
Supplement: Supplementary file 1 — ma3c01083_si_001.pdf [file ma3c01083_si_001.pdf]

Supporting Information

Benzene tetraamide, a covalent-supramolecular dual motif in dynamic covalent polymer networks

Huiyi Zhang,[1] Annemiek van Hertrooij,[1] Tobias Schnitzer,[1] Yinjun Chen,[1] Soumabrata Majumdar,[1] Rolf A.T.M. van Benthem,[2] Rint P. Sijbesma[1,*] and Johan P.A. Heuts[1,*]

[1] *Institute for Complex Molecular Systems & Laboratory of Macromolecular and Organic Chemistry, Department of Chemical Engineering & Chemistry, Eindhoven University of Technology, PO Box 513, 5600 MB Eindhoven, The Netherlands.*

[2] *Laboratory of Physical Chemistry, Department of Chemical Engineering & Chemistry, Eindhoven University of Technology, PO Box 513, 5600 MB Eindhoven, The Netherlands and PTX-C, Shell technology Center Amsterdam, Grasweg 31, 1031 HW Amsterdam*

Corresponding authors: r.p.sijbesma@tue.nl, j.p.a.heuts@tue.nl

Table of Contents

1. Determination of relaxation spectra from stress relaxation data 2
2. Results 3
   2.1 Density Functional Theory (DFT) calculations of tetraamide monomers and dimers 3
   2.2 Additional experimental results related to PMDA and ODPA-based networks 8
   2.3 Additional experimental results related to branched PMDA-based and ODPA-based polyamide 9
   2.4 Additional FT-IR spectra 11
3. References 12

1. Determination of relaxation spectra from stress relaxation data

A generalized Maxwell model (Eq S1) was used to analyze the stress relaxation data and identify the relaxation modes.

$$\sigma(t) = \sigma_e + \int_0^{+\infty} \sigma(\tau) \exp\left(-\frac{t}{\tau}\right) d\tau \qquad \text{(S1)}$$

In this equation, σ(t) is the stress, σ_e is the equilibrium stress, τ is the characteristic relaxation time and σ(τ) is the distribution function of relaxation times. The analysis on stress relaxation data was performed by generating the corresponding relaxation spectra using method, previously reported by Kontogiorgos and group.[1] The method involves the use of Hansen's algorithms that involve L-curve criterion for the determination of optimal regularization parameter and Tikhonov regularization to generate the spectrum in MATLAB.

MATLAB script:

```
Impfile=importdata('File_name.txt');
[A,sp]=discr(1e-4,1e8,[Impfile(:,1)]);
[U,s,V]=csvd(A);
[reg_corner,rho,eta,reg_param]=l_curve(U,s,[Impfile(:,2)],'Tikh');
n=size(V,1);
x_0=zeros(n,1);
[x_lambda,rho,eta] = tikhonov(U,s,V,[PMDA22(:,2)],reg_corner,x_0);
semilogx(sp,x_lambda);
T=table(sp,x_lambda);
writetable (T,'output_file_name.txt');
type output_file_name.txt;
```

The given script generates a spectrum with a relaxation time range of 10^{-4} s to 10^{8} s. The input file format is .txt with two columns (first column representing time and second column representing stress). The given script generates the relaxation spectra as the output and also exports the data points in a .txt file.

2. Results

2.1 Density Functional Theory (DFT) calculations of tetraamide monomers and dimers

Computational analysis of the monomers and dimers were performed using the Schrödinger Maestro Suite 2021-2. The input structures were generated using the built-in 2D structure generator followed by geometry minimization with Macromodel (OPLS4 force field, vacuum). The dimer structures of the non-ether bridged tetra amides were oriented such that linear hydrogen bonds along the helical screw axes are formed. For the ODPA-based tetraamide, no such geometry could be obtained due to steric restrictions, thus a conformational search (OPLS4 force field, vacuum, 5000 steps) was performed and the lowest energy structure was used as starting structure. Geometry optimization of the structures was performed using Jaguar at the B3-LYP-D3/6-311G+** level of theory (vacuum). Frequency calculations on the optimized structures yielded no imaginary frequencies, indicating stationary points on the potential energy surface. The binding energies of the dimers were calculated by subtraction of the total free energies of the corresponding monomers from that of the dimer.

	Total Free Energy (a.u.)	Zero Point Energy (kcal/mol)	Enthalpy (kcal/mol)	Entropy (cal/(mol·K))
PMDA Monomer	–1064.381	201.484	14.021	150.810
PMDA Dimer	2128.797	405.410	29.885	258.283
Monomer	–1370.663	255.237	170.699	18.178
Dimer	–2741.365	512.445	298.317	36.039

Dimerization Energy for PMDA dimer: –21.5 kcal/mol

Dimerization Energy for ODPA dimer: –25.1 kcal/mol

PMDA Monomer:

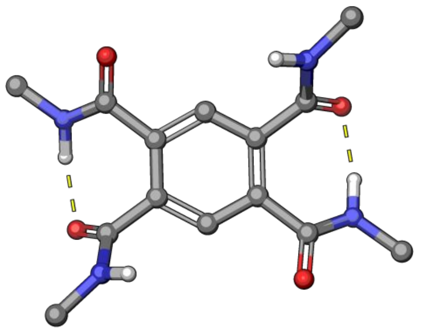

xyz coordinates:

C	-0.26600	-0.20020	-0.90130
C	-0.49770	0.97230	-0.15660
C	-1.70330	1.10750	0.53460
C	-2.71850	0.14980	0.50320
C	-2.47970	-1.03040	-0.22720
C	-1.26090	-1.17960	-0.89180
C	0.98900	-0.60180	-1.66210
C	-3.45980	-2.17180	-0.35880
C	0.47040	2.12640	-0.04720
C	-3.92410	0.50010	1.36290
N	-2.95920	-3.38720	-0.03020
O	-4.60740	-2.01560	-0.77410
C	-3.72260	-4.62160	-0.15160
N	-5.13780	0.08260	0.95360
O	-3.76340	1.18760	2.36970
C	-6.32780	0.40250	1.72600
N	1.68580	0.36460	-2.29120
O	1.30100	-1.78980	-1.72000
C	2.88390	0.03740	-3.04770
N	0.76670	2.49610	1.22230
O	0.91810	2.71100	-1.03280
C	1.62250	3.62990	1.54360
H	-1.88290	1.99240	1.13210
H	-1.05420	-2.09310	-1.43500
H	-2.02380	-3.43080	0.34400
H	-4.59880	-4.42430	-0.76660
H	-4.05330	-4.97900	0.82790
H	-3.11240	-5.39190	-0.62750
H	-5.20880	-0.52010	0.13900
H	-6.45100	1.48460	1.81510
H	-7.19470	-0.01980	1.21810
H	-6.26250	-0.00980	2.73650
H	1.40910	1.33500	-2.17420
H	3.64470	-0.41240	-2.40380
H	2.65530	-0.67710	-3.84220
H	3.27760	0.95370	-3.48710
H	0.39130	1.94370	1.97770
H	2.59340	3.29540	1.91990
H	1.77840	4.20940	0.63560
H	1.14330	4.25950	2.29630

PMDA Dimer:

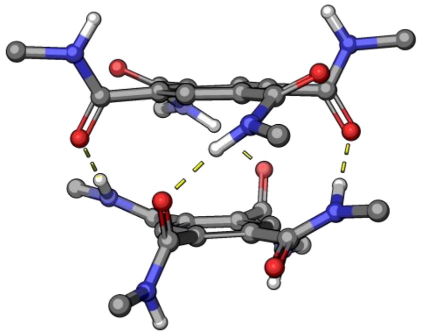

xyz-coordinates:

C	0.01710	0.10840	0.15850
C	-0.77570	1.26350	0.18840
C	-2.16440	1.15340	0.19130
C	-2.79720	-0.09210	0.16310
C	-2.00410	-1.24750	0.16690
C	-0.61550	-1.13730	0.16540
C	1.52160	0.23810	0.15480
C	-2.59460	-2.63440	0.07280
C	-0.18590	2.65210	0.11830
C	-4.30190	-0.22180	0.16380
N	-2.55250	-3.36220	1.20880
O	-2.98630	-3.09780	-0.99820
C	-3.18930	-4.67060	1.28580
N	-4.96730	0.78950	-0.43220
O	-4.83850	-1.20140	0.68330
C	-6.41310	0.80380	-0.58300
N	2.18480	-0.76120	-0.46300
O	2.06040	1.20780	0.69070
C	3.62990	-0.77130	-0.62110
N	-0.22380	3.35850	1.26780
O	0.20170	3.13580	-0.94520
C	0.41290	4.66550	1.36680
H	-2.75830	2.05800	0.19250
H	-0.02180	-2.04150	0.14640
H	-2.40650	-2.85140	2.06580
H	-4.27970	-4.57810	1.29290
H	-2.89720	-5.26260	0.41910
H	-2.85810	-5.17420	2.19420
H	-4.44760	1.46410	-0.99450
H	-6.82770	1.72690	-0.16900
H	-6.67540	0.74250	-1.64230
H	-6.83430	-0.04760	-0.05130
H	1.66310	-1.42490	-1.03580
H	4.04710	-1.70220	-0.22810
H	3.88690	-0.68820	-1.68020
H	4.05310	0.06940	-0.07420
H	-0.36650	2.83170	2.11570
H	1.50340	4.57320	1.36810
H	0.11740	5.27340	0.51230
H	0.08490	5.15200	2.28570
C	-0.47540	-1.00880	-3.41740
C	-0.01040	0.31290	-3.38830
C	-0.94570	1.35090	-3.39360
C	-2.31500	1.09570	-3.39250
C	-2.77980	-0.22630	-3.38750
C	-1.84480	-1.26390	-3.41740
C	0.45580	-2.19620	-3.35040
C	-4.26580	-0.49330	-3.38640
C	1.47590	0.57970	-3.38770
C	-3.24570	2.28130	-3.29780
N	-4.64510	-1.64250	-2.79100
O	-5.03920	0.31300	-3.90610
C	-6.03740	-2.03300	-2.64200
N	-3.39480	2.99570	-4.43310
O	-3.74400	2.62650	-2.22630
C	-4.34870	4.09450	-4.50920
N	0.60090	-2.88610	-4.50160
O	0.95750	-2.56410	-2.28840
C	1.55480	-3.98270	-4.60470
N	1.85670	1.71800	-2.77200
O	2.24780	-0.21650	-3.92450
C	3.24930	2.10670	-2.62090
H	-0.60780	2.37870	-3.37560
H	-2.18250	-2.29180	-3.41840
H	-3.96850	-2.15870	-2.22860
H	-6.19570	-3.03360	-3.05260
H	-6.30950	-2.03860	-1.58360
H	-6.66500	-1.32290	-3.17770
H	-3.11990	2.54170	-5.29060
H	-4.15700	4.66960	-5.41540
H	-5.37790	3.72250	-4.51990
H	-4.22210	4.73940	-3.64020
H	0.32330	-2.41340	-5.34810
H	1.36010	-4.53820	-5.52240
H	2.58390	-3.61020	-4.61120
H	1.43150	-4.64600	-3.74940
H	1.18170	2.22300	-2.19740
H	3.52570	2.09200	-1.56360
H	3.87530	1.40780	-3.17280
H	3.40490	3.11540	-3.01270

Monomer:

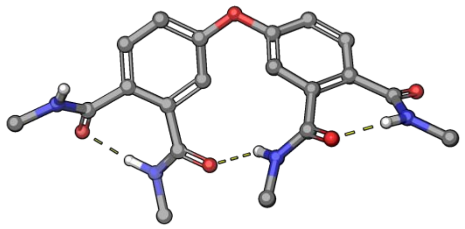

xyz-coordinates:

C	1.49890	4.82080	-2.12630
C	0.70210	3.70130	-1.91890
C	1.06300	2.50640	-2.52350
C	2.21950	2.43140	-3.29010
C	2.98720	3.56860	-3.55000
C	2.63930	4.79390	-2.94080
C	-0.62710	-1.53790	-4.31090
C	-0.45360	-1.10320	-5.63530
C	-0.08360	0.24150	-5.85500
C	0.08250	1.08140	-4.75580
C	-0.02560	0.61350	-3.45040
C	-0.40900	-0.70510	-3.22140
O	0.23530	1.40270	-2.35620
C	4.10880	3.36450	-4.54510
C	3.31230	6.14650	-3.11220
C	0.32970	0.90280	-7.16110
C	-0.70960	-2.09880	-6.73190
N	-0.13810	-3.31770	-6.56040
O	-1.42860	-1.85860	-7.70320
C	-0.33410	-4.39900	-7.51730
N	-0.35080	0.60620	-8.27800
O	1.25480	1.72500	-7.14790
C	0.01070	1.20450	-9.55390
N	3.73070	2.79640	-5.71060
O	5.28420	3.65470	-4.30890
C	4.70420	2.49800	-6.75110
N	4.64020	6.16890	-3.35200
O	2.65210	7.17530	-2.97870
C	5.33290	7.43450	-3.52750
H	1.21890	5.77150	-1.69150
H	-0.19880	3.75100	-1.32000
H	2.49840	1.49060	-3.74490
H	-0.94250	-2.55880	-4.12970
H	0.36690	2.10460	-4.94380
H	-0.51940	-1.06250	-2.20540
H	0.54870	-3.42770	-5.83150
H	-1.37290	-4.40250	-7.84580
H	0.30290	-4.27750	-8.39860
H	-0.10280	-5.34860	-7.03420
H	-1.03880	-0.14260	-8.24160
H	0.00170	2.29360	-9.47830
H	1.01310	0.89720	-9.86570
H	-0.71350	0.88650	-10.30320
H	2.75900	2.58290	-5.91100
H	5.45410	1.78990	-6.38940
H	4.16900	2.06760	-7.59620
H	5.22370	3.40590	-7.06700
H	5.13800	5.29650	-3.50820
H	5.23010	8.05860	-2.63630
H	6.38860	7.22940	-3.70550
H	4.92430	7.99460	-4.37340

Dimer:

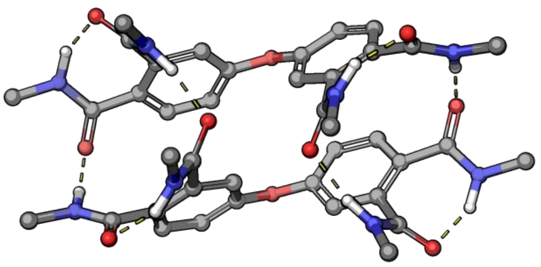

xyz-coordinates:

C	-2.91870	1.41270	-1.26950
C	-1.90530	1.29490	-2.20660
C	-1.26880	0.06720	-2.36570
C	-1.64480	-1.02340	-1.59280
C	-2.66900	-0.90590	-0.64880
C	-3.33170	0.32610	-0.48640
C	2.16580	-2.46650	-4.37040
C	1.34800	-3.42190	-4.98690
C	-0.04880	-3.22500	-4.95650
C	-0.57290	-2.08110	-4.34990
C	0.26860	-1.13500	-3.77680
C	1.64470	-1.32590	-3.78000
O	-0.20660	0.04610	-3.24370
C	-2.85470	-2.13320	0.21460
C	-4.48600	0.54720	0.45420
C	-1.06370	-4.18460	-5.53470
C	2.08760	-4.60350	-5.56490
N	1.58930	-5.20500	-6.65650
O	3.15830	-4.97030	-5.05380
C	2.26240	-6.34580	-7.25580
N	-1.98830	-4.61490	-4.65900
O	-1.06800	-4.50890	-6.72800
C	-3.04910	-5.52190	-5.06720
N	-4.10240	-2.55750	0.46150
O	-1.85450	-2.73390	0.63650
C	-4.34540	-3.70710	1.31720
N	-4.39290	1.63800	1.23650
O	-5.47830	-0.19410	0.46900
C	-5.46220	1.99740	2.15340
H	-3.40700	2.36990	-1.13960
H	-1.58930	2.13930	-2.80600
H	-1.16050	-1.98090	-1.71120
H	3.23150	-2.64910	-4.34780
H	-1.64420	-1.93210	-4.31810
H	2.28590	-0.59570	-3.30390
H	0.68240	-4.90740	-7.01480
H	2.21040	-7.22660	-6.60800
H	1.77850	-6.57520	-8.20470
H	3.31560	-6.11690	-7.42890
H	-1.84850	-4.43050	-3.66450
H	-2.66030	-6.52170	-5.28500
H	-3.77860	-5.58760	-4.26000
H	-3.53590	-5.14790	-5.96900
H	-4.86690	-1.91320	0.25570
H	-3.65000	-4.50710	1.06100
H	-4.20780	-3.46110	2.37600
H	-5.36900	-4.04910	1.16590
H	-3.49660	2.11980	1.31280
H	-6.41190	2.06750	1.61990
H	-5.57880	1.25140	2.94580
H	-5.22460	2.96330	2.59900
C	0.36700	1.35510	0.58210
C	1.24980	0.58410	-0.15780
C	1.58580	-0.68440	0.29840
C	1.03640	-1.17480	1.47710
C	0.15730	-0.39390	2.23100
C	-0.17750	0.90400	1.79100
C	3.34030	-4.72440	-1.58750
C	2.09500	-5.36500	-1.64620
C	0.95010	-4.63710	-1.26830
C	1.08150	-3.31810	-0.82410
C	2.33110	-2.71550	-0.76200
C	3.47010	-3.41800	-1.14560
O	2.52250	-1.39250	-0.42730
C	-0.38000	-1.03890	3.48790
C	-1.12980	1.87000	2.45230
C	-0.48230	-5.09450	-1.42660
C	2.07600	-6.80760	-2.07660
N	2.75890	-7.08400	-3.20240
O	1.51570	-7.68880	-1.41040
C	2.86450	-8.44580	-3.70020
N	-0.81420	-6.31790	-0.98950
O	-1.31490	-4.31760	-1.91880
C	-2.16540	-6.82870	-1.15110
N	-1.00290	-2.21340	3.29170
O	-0.21870	-0.54570	4.61030
C	-1.54840	-2.97150	4.40640
N	-1.20410	1.88630	3.79250
O	-1.79130	2.65730	1.75620
C	-2.10510	2.79570	4.48170
H	0.06490	2.32890	0.22130
H	1.66270	0.94200	-1.09190
H	1.28810	-2.17370	1.80850
H	4.22530	-5.26710	-1.89410
H	0.19200	-2.78390	-0.52610
H	4.43500	-2.92860	-1.10610
H	3.05660	-6.31600	-3.80500
H	1.88910	-8.83800	-4.00480
H	3.53840	-8.44850	-4.55670
H	3.26050	-9.10390	-2.92450
H	-0.05300	-6.96540	-0.78190
H	-2.88400	-6.05280	-0.88450
H	-2.36230	-7.13590	-2.18430
H	-2.29720	-7.69020	-0.49690
H	-1.22610	-2.50060	2.33770
H	-2.36510	-2.43070	4.89410
H	-1.92050	-3.92290	4.02690
H	-0.77560	-3.15620	5.15480
H	-0.68500	1.18900	4.32530
H	-1.89630	2.74780	5.54990
H	-1.95800	3.81730	4.12630
H	-3.15230	2.52660	4.31180

2.2 Additional experimental results related to PMDA and ODPA-based networks

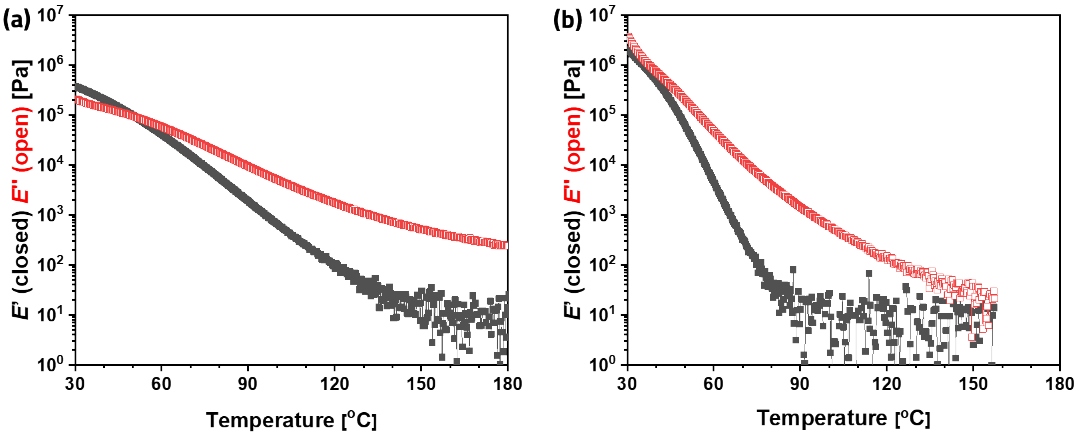

Figure S1. Dynamic mechanical thermal analysis of (a) PMDA-based polyimide from 30 to 180 °C and (b) ODPA-based polyimide from 30 to 160 °C at the rate of 3 °C·min^{-1} (strain of 1 % and frequency of 1 Hz).

Table S1 Gel content and swelling ratios of PMDA-based and ODPA-based networks in THF or methanol/THF

Network	THF		Methanol/THF	
	Swelling ratio	Gel content	Swelling ratio	Gel content
**PMDA**	1.38 ± 0.02	0.97 ± 0.02	1.55 ± 0.04	0.92 ± 0.02
**ODPA**	2.44 ± 0.04	0.86 ± 0.02	2.41 ± 0.11	0.85 ± 0.03

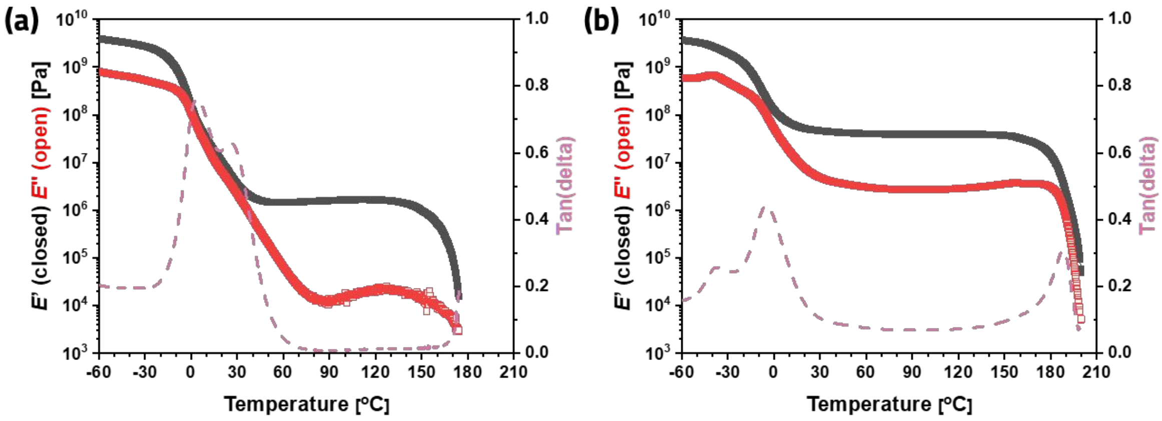

Figure S2. Dynamic mechanical thermal analysis of (a) ODP4A and (b) B4A polyamide network from -60 °C to 180 °C for ODPA network and 200 °C for PMDA network at a heating rate of 3 °C·min^{-1} with a frequency of 1 Hz.

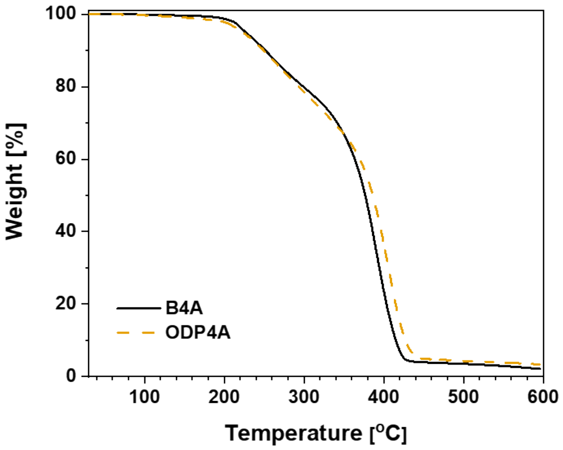

Figure S3. TGA thermograms of the different covalent adaptable B4A-based and ODP4A-based networks obtained using a heating ramp from 30 to 600°C at a rate of 10°C·min^{-1} under N_2 flow.

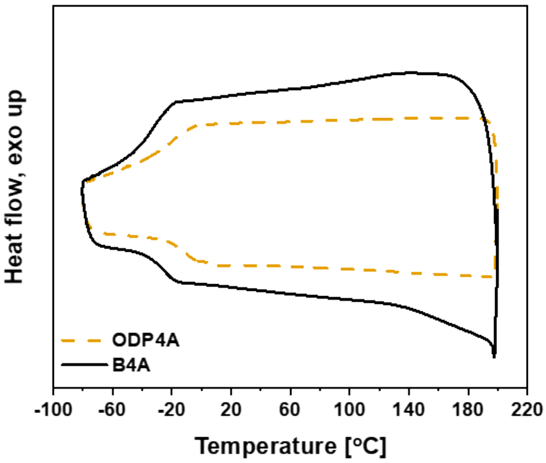

Figure S4. DSC curves of B4A-based and ODP4A-based networks from -80 to 200°C at a ramping rate of 20°C·min^{-1} under nitrogen atmosphere.

2.3 Additional experimental results related to branched B4A-based and ODP4A-based polyamides

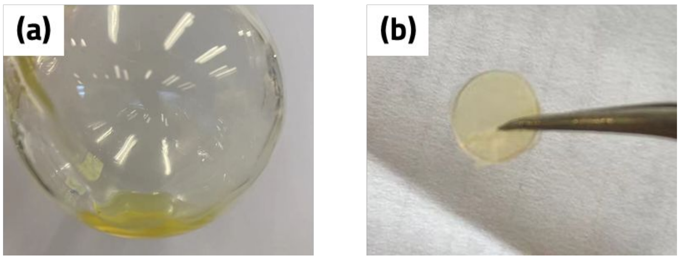

Figure S5. Photos of (a) branched ODP4A polyamide and (b) compression molded round disk sample of branched B4A polyamide.

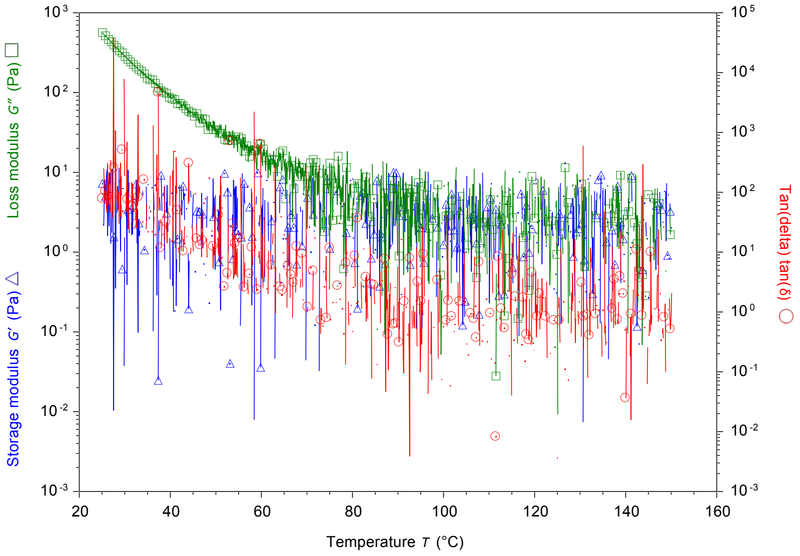

Figure S6. Temperature sweep rheology of branched ODP4A-based polyamide.

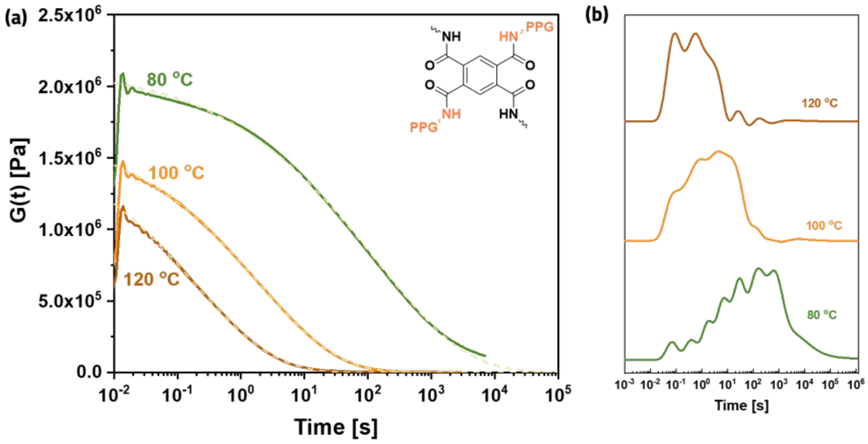

Figure S7. (a) Stress relaxation curves (solid line) and the fitted curved (dash line) at different temperatures from 80 to 120 °C using stretched exponential decay function (**eq 3**, fitted from t = 0.1s) of B4A-based polyamide brush and (b) the corresponding relaxation spectra using a generalized Maxwell model.

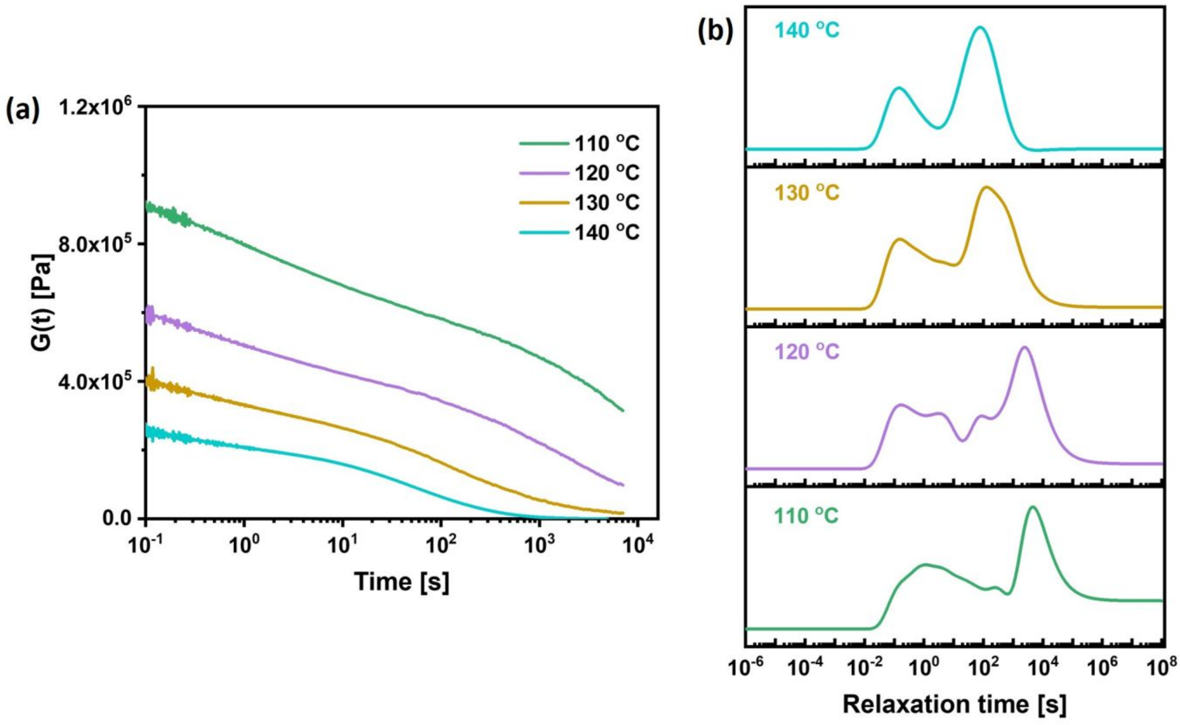

Figure S8. (a) Stress relaxation curves for our previously published B4A-based poly(tetrahydrofuran) networks,[2] applied step strain = 1%, (b) the corresponding relaxation spectra using a generalized Maxwell model.

2.4 Additional FT-IR spectra

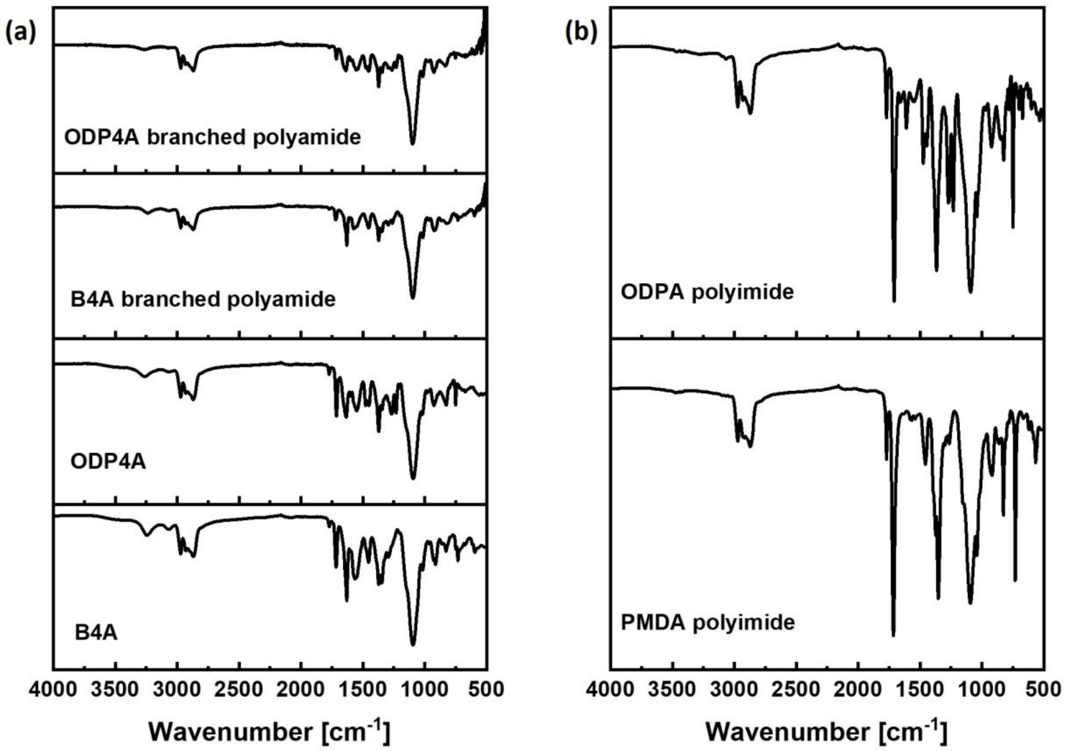

Figure S9. FT-IR spectra of (a) B4A and ODP4A branched polyamides and networks and (b) the precursor polyimides.

3. References

(1) Kontogiorgos, V. Calculation of relaxation spectra from stress relaxation measurements. *Biopolymers* (IntechOpen, 2010), Chap 25.

(2) Chen, Y.; Zhang, H.; Majumdar, S.; Van Benthem, R.A.T.M.; Heuts, J.P.A.; Sijbesma, R.P. Dynamic Polyamide Networks via Amide-Imide Exchange. *Macromolecules* **2021**, *54*, 9703-9711.
